# Global DNA Barcoding of *Sigara* (Hemiptera: Corixidae) Reveals Cryptic Species Formation and Climatic-Niche Divergence

**DOI:** 10.3390/insects17080876

**Published:** 2026-08-21

**Authors:** Zonglei Liang, Kexin Ren, Bingjiao Sun, Tongyin Xie

**Affiliations:** 1 College of Plant Protection, Northeast Agricultural University, Harbin 150030, China; zonglei@neau.edu.cn (Z.L.); renkexinup@163.com (K.R.); 2 China National Environmental Monitoring Centre, Beijing 100012, China

**Keywords:** DNA barcoding, cryptic species, *COI*, species delimitation

## Abstract

The genus *Sigara* Fabricius, 1775 (Hemiptera: Corixidae) is an important freshwater bioindicator, but its conservative morphology complicates traditional species identification. This study combines DNA barcoding, traditional taxonomy, and climatic factors within an integrative framework to provide new insights into species delimitation. We analyzed cytochrome *c* oxidase subunit I (*COI*) sequences from 501 *Sigara* specimens collected from 15 countries, including 95 newly generated *COI* barcodes. Our integrated approach included phylogenetic analyses, genetic distance assessments, and ecological niche modeling. The results revealed remarkable diversity, with sequences clustering into 46 Barcode Index Numbers (BINs). *COI* genetic distances showed a pronounced bimodal pattern, with most intraspecific divergences below 1% and interspecific divergences mainly ranging from 8–16%, while the few intermediate divergences (4–8%) suggested potential cryptic diversity or recent species divergence. Most morphologically defined species corresponded to distinct molecular lineages, supporting traditional taxonomy, whereas some morphospecies exhibited genetic discordance potentially associated with species divergence. Integrative analyses revealed that climatic factors, particularly isothermality and seasonal temperature and precipitation, are major drivers of *Sigara* MOTU distributions, highlighting the important role of climate in shaping their geographic patterns. These findings indicate that although *COI* barcodes are not sufficient to define the species boundaries of *Sigara* alone, they provide a key entry point for the accurate division of species, which in turn promotes the development of molecular tools for freshwater ecological monitoring and conservation.

## Simple Summary

Identifying Corixidae is challenging because many species have similar appearances and hidden diversity. Our study investigated the globally distributed genus *Sigara* using DNA barcoding and ecological analyses. By examining 501 *COI* sequences from 15 countries, we revealed remarkable genetic diversity within this genus and, in combination with climatic factors, analyzed the factors influencing the distribution of its lineages.

## 1. Introduction

**Figure 1:**
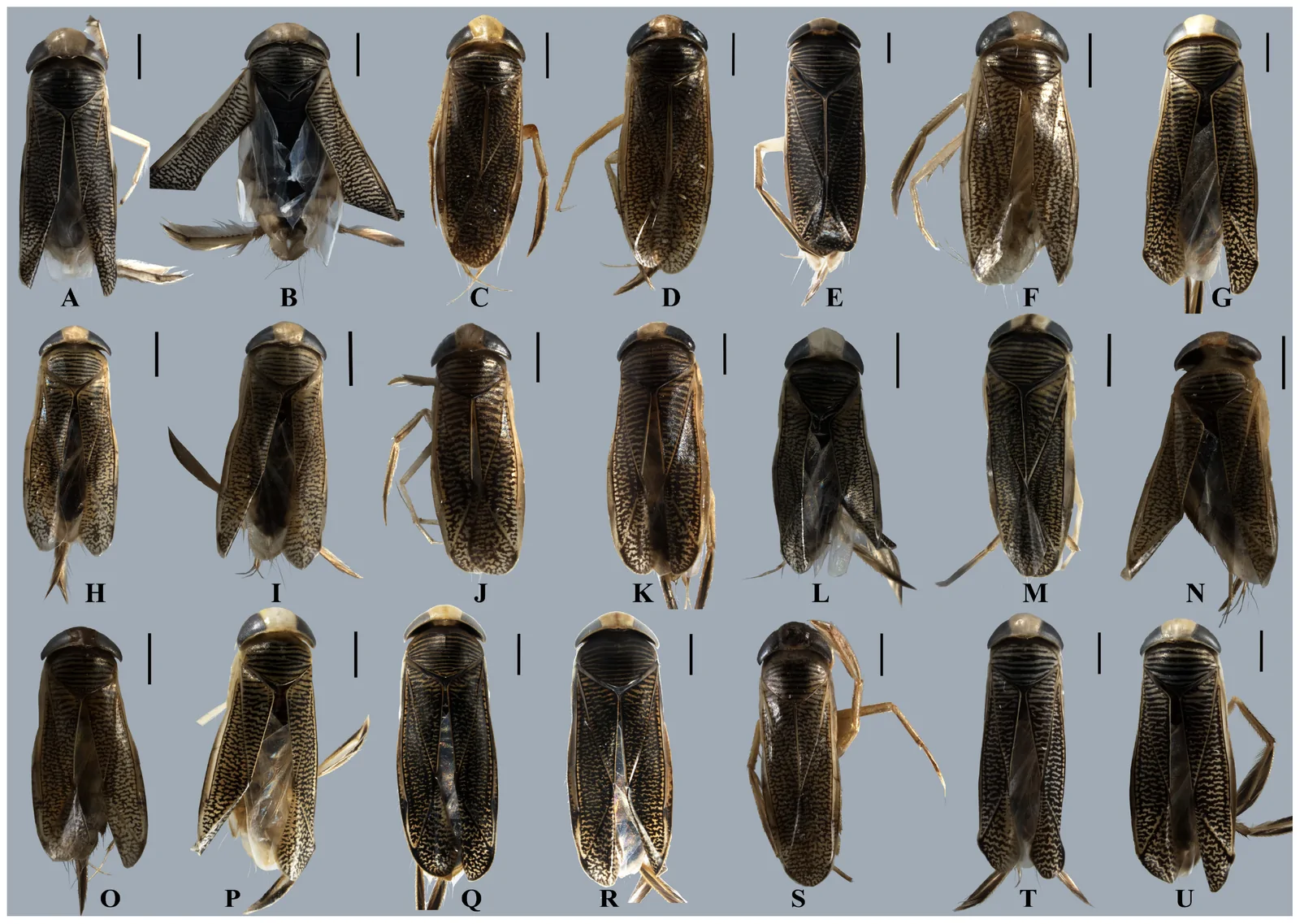
Dorsal habitus photographs of *Sigara* in China. (**A**) *Sigara assimilis* (Fieber, 1848) ♂; (**B**) *S. assimilis* (Fieber, 1848) ♀; (**C**) *S. bellula* (Horváth, 1879) ♂; (**D**) *S. bellula* (Horváth, 1879) ♀; (**E**) *S. distincta* (Fieber, 1848) ♀; (**F**) *S. distorta* (Distant, 1911) ♂; (**G**) *S. falleni* (Fieber, 1848) ♂; (**H**) *S. gaginae* Jaczewski, 1960 ♂; (**I**) *S. gaginae* Jaczewski, 1960 ♀; (**J**) *S. kerzhneri* Jaczewski, 1963 ♂; (**K**) *S. kerzhneri* Jaczewski, 1963 ♀; (**L**) *S. lateralis* (Leach, 1817) ♂; (**M**) *S. lateralis* (Leach, 1817) ♀; (**N**) *S. septemlineata* (Paiva, 1918) ♂; (**O**) *S. septemlineata* (Paiva, 1918) ♀; (**P**) *S. striata* (Linnaeus, 1758) ♂; (**Q**) *S. substriata* (Uhler, 1897) ♂; (**R**) *S. substriata* (Uhler, 1897) ♀; (**S**) *S. takahashii* Hungerford, 1940 ♂; (**T**) *S. weymarni* Hungerford, 1940 ♂; (**U**) *S. weymarni* Hungerford, 1940 ♀; Scale bars = 1 mm.

The genus *Sigara* (Heteroptera: Nepomorpha: Corixoidea: Corixidae) comprises small to medium-sized (4.5-8 mm) aquatic bugs, which are known as water boatmen and are distributed worldwide [1,2,3]. The genus *Sigara* comprises approximately 188 described species worldwide (Table S1) including 22 recorded species (Figure 1A–U) in China [4,5]. The superfamily Corixoidea is one of the largest superfamilies of aquatic bugs (Hemiptera: Heteroptera: Nepomorpha) and includes more than 600 extant species [6,7]. *Sigara* plays an indispensable ecological role in maintaining the stability of freshwater ecosystems and driving material cycling, precisely due to the widespread distribution and habitat stability of this group in freshwater ecosystems [8]. Moreover, owing to its high sensitivity to changes in water quality, this genus occupies an important position in aquatic biological communities and is frequently employed as a bioindicator for freshwater ecological monitoring [9,10]. Consequently, accurate and rapid species identification of *Sigara* is not only a fundamental task in taxonomic research but also a critical prerequisite for studies in aquatic biology, biodiversity conservation and related fields [10,11,12].

The genus *Sigara* is taxonomically complex, divided into several subgenera such as *Antisigara*, *Pseudovermicorixa*, *Retrocorixa*, *Sigara*, *Subsigara*, *Tropocorixa*, *Vermicorixa* [5]. Members of this genus are significant components of freshwater ecosystems, exhibiting a wide range of ecological and morphological traits [13,14]. Key distinguishing features for species identification include body size, colour patterns, the shape of the male right paramere, the number of pegs on the male fore tarsus, the size and structure of the strigil, and the morphology of the mesepimeron. Many species within the genus are morphologically very similar, often requiring examination of male genital structures for reliable identification [15,16]. It relies on both specimen integrity and the taxonomists’ expertise and experience. For non-specialists in ecological research, it is often very difficult to accurately identify the *Sigara* species based on traditional morphological methods, which restricts the in-depth development of related fields [17]. Intraspecific variation within the genus may also result in inaccurate species identification [18,19,20].

Recent molecular phylogenetic studies of Corixidae in the Palearctic region showed that the error rate of morphological identification was 20–40% in some groups [7]. Based on the sequencing of mitochondrial genomes of *S. lateralis* and other species, phylogenetic analysis was carried out. The results showed that *S. lateralis* and *S. septemlineata* were sister groups, and the relationship between them was the closest [3]. In addition, studies have found that *S. potamius* and *S. limnochares* in New Zealand are grouped into the same species based on DNA analysis [14], and other researchers have found that *S. distincta* in Europe has two highly differentiated genetic lineages [21]. Molecular data reveal two typical contradictions: first, species that exhibit consistent morphology but genetic divergences exceeding 5%; second, morphologically divergent taxa exhibiting low genetic divergence, which may lead to misidentification [7]. These findings highlight the need for an integrative taxonomic approach that combines morphology with molecular evidence for reliable species delimitation in *Sigara*.

DNA barcoding technology realizes rapid molecular identification of species through standardized gene fragments (such as *COI*), and effectively reveals cryptic species based on genetic distance threshold, injecting new impetus into taxonomy [22,23,24]. However, mitochondrial molecular data cannot fully reflect the biological characteristics of species due to the interference of pseudogenes, intraspecific variation and sample size [25]. Therefore, it is an inevitable trend to solve the identification of related species by combining DNA barcoding with traditional morphological classification and carrying out comprehensive taxonomy [26,27].

To address these gaps, this study aims to integrate a global *COI* DNA barcode dataset for the genus *Sigara* and investigate its molecular diversity and broad-scale ecological and geographic patterns. Specifically, we aim to: (1) assess the genetic diversity and molecular differentiation of *Sigara*; (2) identify potential cryptic diversity and molecular operational taxonomic units (MOTUs); (3) evaluate the geographic and ecological distribution patterns of *Sigara* and their associations with major environmental gradients. By integrating DNA barcoding with broad-scale ecological niche and environmental analyses, this study provides a molecular framework for uncovering hidden diversity in *Sigara* and advances our understanding of its diversity, geographic distribution, and ecological differentiation, thereby contributing to a more comprehensive understanding of the biogeographic patterns of this ecologically important aquatic insect.

## 2. Materials and Methods

Quantitative sampling was conducted from 2024 to 2026 using aquatic nets. The experimental insects were mainly collected from Gansu Province, Hebei Province, Heilongjiang Province, Hubei Province, Liaoning Province, Ningxia Hui Autonomous Region, Qinghai Province, Shaanxi Province and Xinjiang Uygur Autonomous Region in Mainland China. The samples were stored in 75% ethanol in the dark to 4 °C [28,29,30,31]. 

Morphological identification of the specimens was performed under a stereomicroscope (Carl Zeiss Microscopy GmbH, Jena, Germany), with reference to relevant taxonomic publications [32]. For molecular sequencing, DNA was extracted from three legs taken from the same side of each specimen using the Ezup Column Animal Genomic DNA Extraction Kit (Sangon Biotech, Shanghai, China). The extraction was performed following the established protocol, which included lysis, column purification, and other relevant steps. DNA purity was assessed using a spectrophotometer (Tiangen Biotech CO., LTD, Beijing, China) [33,34]. Samples that met quality standards were subjected to PCR amplification of the *COI* gene using the universal primers LCO1490 and HCO2198 [35,36]. Following electrophoresis detection, the amplified products were submitted for sequencing. The obtained sequences were subsequently trimmed and aligned using Geneious Prime v2025.0.2. After quality trimming, the retained barcode sequences were 504–658 bp [37]. Sequences with internal stop codons or obvious anomalies were excluded.

The redundant sequences of *Sigara* were retrieved from the Barcode of Life Data System (BOLD) [38]. After excluding sequences containing stop codons, BOLD markers with quality problems, or sequences lacking effective BIN classification information, the *COI* sequence from BOLD was retained for the study of *Sigara*. A total of 406 sequences were retrieved, 95 new sequences were generated, and a dataset containing 501 sequences was formed, named “Global COI barcodes of *Sigara* (DS-SIGARA)” (data acquisition time was 29 March 2026).

In this study, multiple sequence alignment was performed using the MUSCLE algorithm implemented in MEGA12 [39,40]. Genetic distances were calculated using the p-distance model, and an unrooted Neighbor-Joining (NJ) tree was constructed based on the Kimura two-parameter (K2P) model under default parameters [41,42,43]. We assessed the support values of each node via 1000 bootstrap replicates [43]. The K2P distance was adopted to ensure comparability with most DNA barcoding studies of insects.

In addition, the Automatic Barcode Gap Discovery (ABGD) [44] method was used to delimit molecular operational taxonomic units (MOTUs). Pairwise genetic distances were calculated using the Kimura two-parameter (K2P) model, and ABGD was run with the settings: P_min_ = 0.001, P_max_ = 0.1, 10 steps, and X = 1.1.

To investigate the potential ecological niche and geographic distribution patterns of the genus *Sigara*, key environmental factors were collected, including 19 bioclimatic variables [45], aridity index [46], mean annual cloud cover [47], and other variables. All environmental variables were obtained in GeoTIFF (.tif) or NetCDF (.nc) format from databases such as CHELSA, CGIAR-CSI, and EarthEnv. Environmental variables were standardized in R [48], and principal component analysis (PCA) was subsequently used to explore the distribution patterns of samples along environmental gradients.

## 3. Results

### 3.1. Dataset Characteristics and Global Distribution

**Figure 2:**
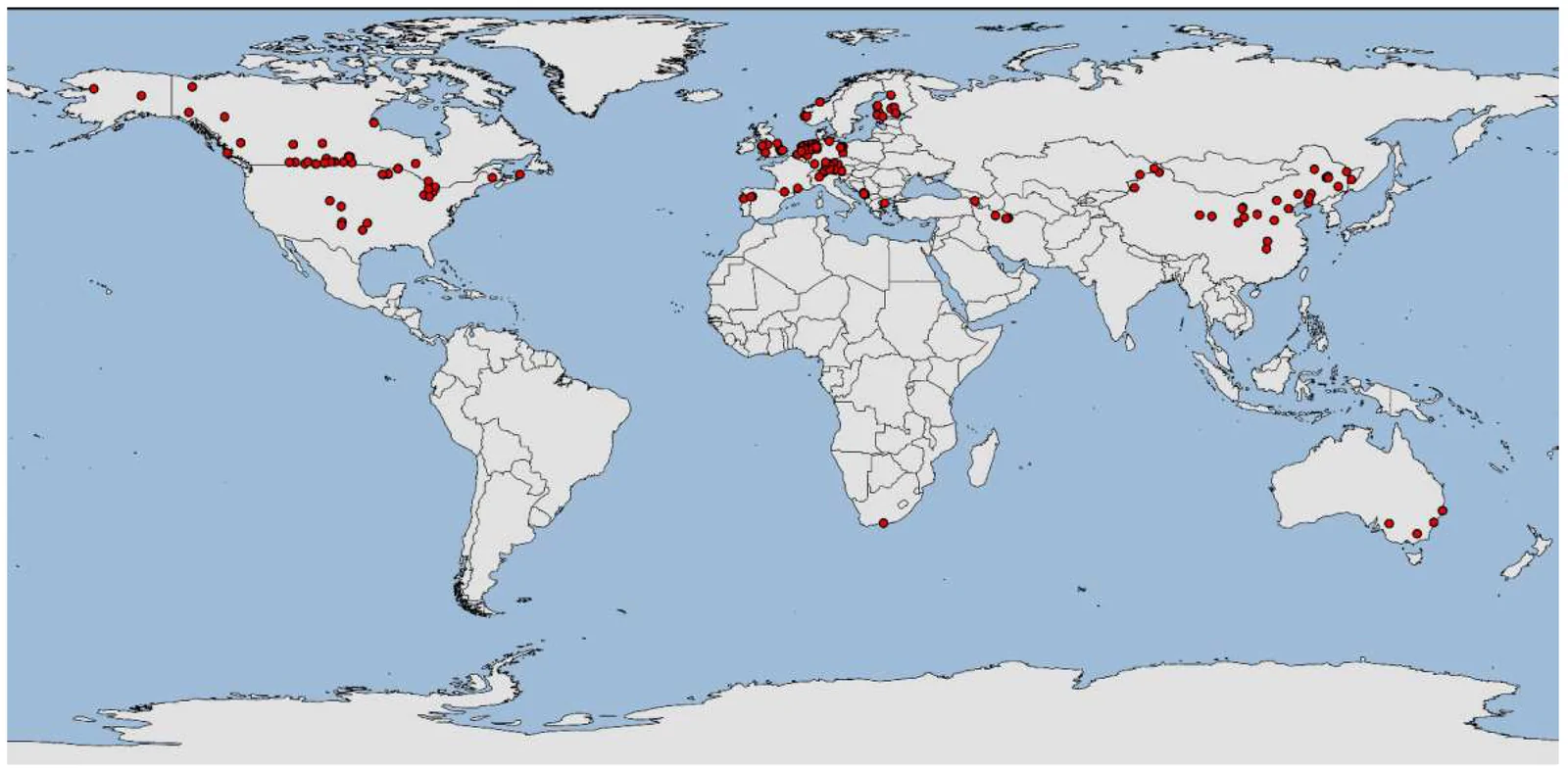
Red markers indicate geographical distribution of *Sigara* specimens used for DNA barcoding in this study.

The dataset comprised a total of 501 sequences, ranging in lengths from 504 bp to 658 bp. Among these, 289 sequences (57.05%) were 658 bp in length, indicating a high level of data completeness. These sequences were classified into 46 BINs, comprising 31 concordant BINs and 15 singleton BINs. The dataset showed a marked geographic sampling imbalance across the six major regions. Western Europe had the highest representation, comprising 170 sequences (33.93%), followed by North America with 129 sequences (25.75%) and East Asia with 98 sequences (19.56%). In contrast, Southern Europe and Northern Europe were poorly represented, accounting for 9.78% (49 sequences) and 5.99% (30 sequences), respectively. Oceania had the lowest coverage, with only 12 sequences (2.39%), all from Australia. This uneven distribution largely reflects differences in regional sampling intensity and highlights potential geographic biases in current barcoding efforts for the genus *Sigara* (Figure 2). The samples cover most biogeographic regions, with regional variation in representation reflecting differences in sampling effort and the distribution of *Sigara*.

### 3.2. Species Clustering

**Figure 3:**
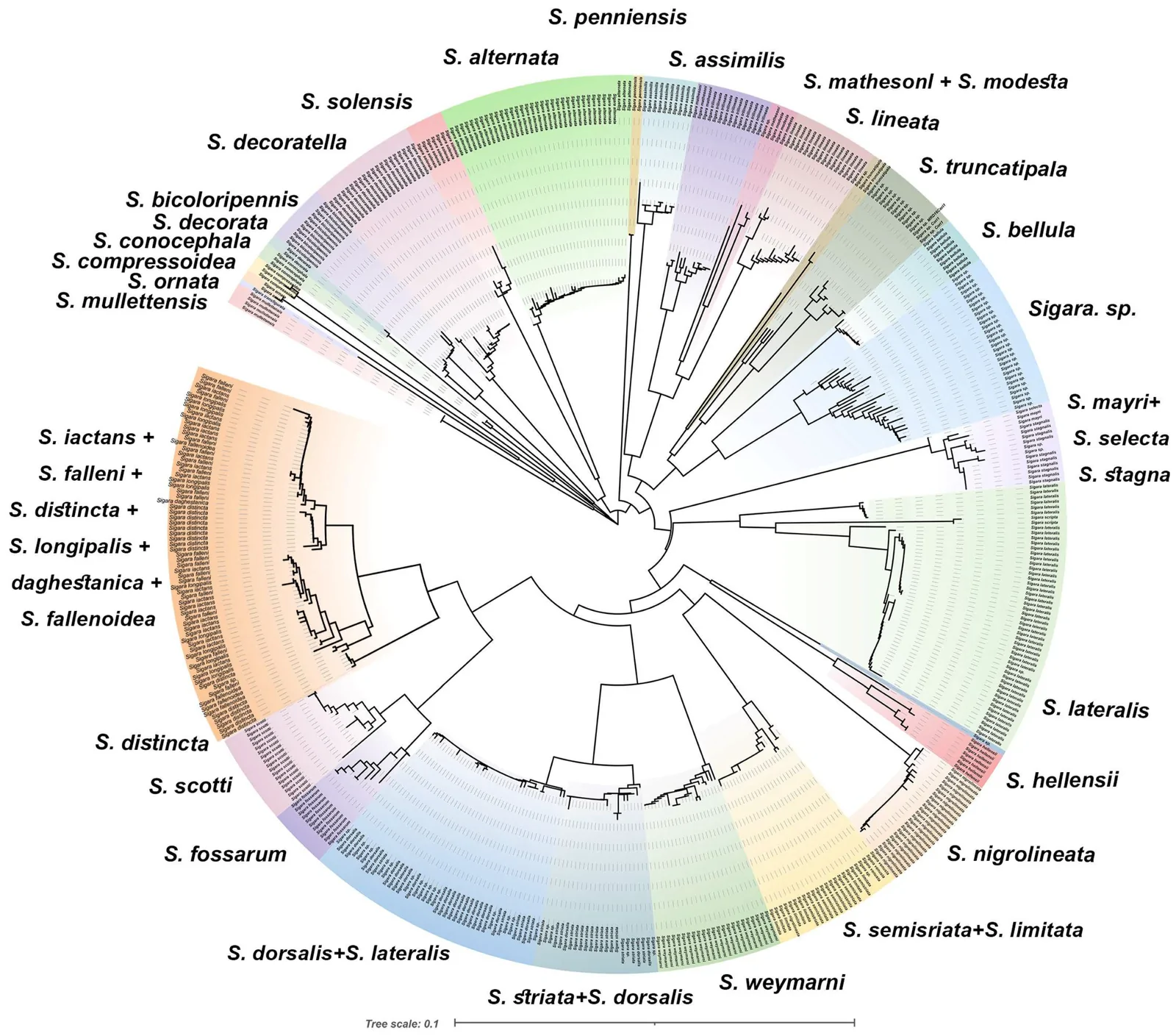
NJ tree constructed based on the *COI* barcodes of *Sigara*.

In this study, 501 DNA barcode sequences were divided into 31 molecular operational taxonomic units (MOTUs) based on the Neighbor-Joining (NJ) phylogenetic tree constructed by *COI* sequences (Figure 3). Furthermore, all newly collected morphospecies that were validated by morphological analysis formed monophyletic groups. Genetic distance analysis revealed that the maximum interspecific genetic distance within the genus *Sigara* was 0.168, and the minimum interspecific genetic distance was 0. Both *S. dorsalis* and *S. striata*, each represented by relatively large numbers of sequences, were split into two MOTUs, with the resulting lineages showing reciprocal genetic admixture. *S. truncatipala* was split into two separate MOTUs. Several taxa, including *S. weymarni*, *S. alternata*, *S. bellula*, *S. hellensii*, *S. lineata*, *S. assimilis*, *S. penniensis*, *S. scotti*, *S. fossarum*, *S. decoratella*, *S. bicoloripennis*, *S. decorata*, *S. conocephala*, *S. compressoidea*, *S. ornata*, *S. mullettensis*, and *S. solensis*, each formed a monophyletic group with clear intraspecific consistency. *S. scotti* and *S. fossarum* formed two independent but closely related sister clades. Notably, *S. selecta*, *S. stagnalis*, and *S. mayri* constituted an independent clade, within which the three taxa were intermingled with each other. Similarly, *S. venusta*, *S. semistriata*, *S. limitata*, and *S. nigrolineata* also clustered into a distinct clade, with their sequences were intermixed within the group. A similar pattern was observed in *S. falleni*, *S. longipalis*, *S. fallenoidea*, *S. distincta*, and *S. iactans*, which also formed a separate but poorly differentiated lineage.

### 3.3. Species Delimitation and Genetic Differentiation Revealed by ABGD Analysis

**Figure 4:**
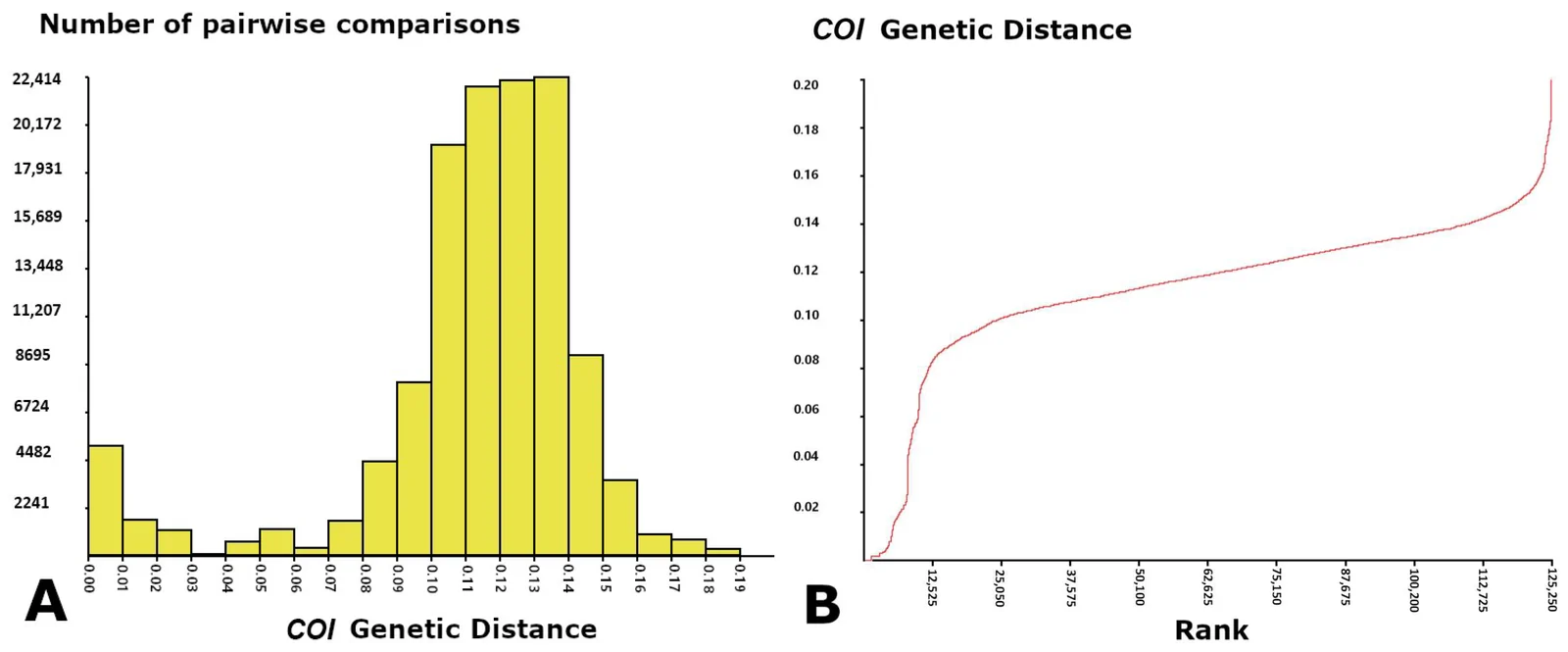
ABGD analysis of *COI* sequences for *Sigara*. (**A**) Distribution of pairwise genetic distances based on the number of comparisons; (**B**) Rank-ordered curve of pairwise genetic distances.

The distribution of pairwise *COI* genetic distances showed a pronounced bimodal pattern (Figure 4A). Most intraspecific comparisons were concentrated below 1%, whereas the majority of interspecific comparisons ranged from approximately 8–16%. The ranked *COI* distance distribution was consistent with the above findings, showing two major divergence levels (Figure 4B). Most comparisons had low genetic distances (0–2%), suggesting stable intraspecific variation, while a few highly divergent comparisons (16–20%) indicated deeply divergent lineages. The few pairwise comparisons with genetic distances of 4–8% may indicate shallow divergence among closely related species, diversity, or taxa requiring further investigation. The clear separation between these two distributions indicates the presence of a distinct barcode gap, suggesting that *COI* provides strong discriminatory power for species identification within this genus. The presence of this gap is a prerequisite for the automatic MOTU delimitation by the ABGD algorithm.

We performed MOTU delimitation using ABGD. The number of MOTUs gradually converged and stabilized at 29 units, though sample sizes were highly unevenly distributed among them (Table S2). Among these, Group 1 (n = 90) and Group 2 (n = 98) were the two largest clusters, together accounting for 37.5% of the total sample size. Nearly one-third of the MOTUs were rare groups, comprising only one or two samples. In contrast, the other groups contained 20–50 sequences each, indicating relatively well-sampled genetic clusters. Among all samples, a total of 16 MOTUs contained only a single species, including *S. mullettensis*, *S. hellensii*, *S. bicoloripennis*, *S. alternata*, *S. compressoidea*, *S. lineata*, *S. decoratella*, *S. solensis*, *S. conocephala*, *S. bellula*, *S. assimilis*, *S. truncatipala*, *S. decorata*, *S. penniensis*, *S. ornata*, and *S. scripta*, accounting for 55.2% of the total MOTUs. This finding preliminarily validated the reliability of the ABGD delimitation results in this study.

However, three species were split into multiple MOTUs. *S. lateralis* was assigned to Groups 1, 4, and 29, *S. truncatipala* to Groups 3 and 5, and *S. nigrolineata* to Groups 16 and 19; each species had at least one single-species MOTU (Groups 4, 29, 3, and 19, respectively). The remaining MOTUs showed complex taxonomic compositions. Group 1 comprised four species (*S. dorsalis*, *S. lateralis*, *S. striata*, and *S. weymarni*); Group 2 included eight species (*S. distincta*, *S. fossarum*, *S. falleni*, *S. scotti*, *S. fallenoidea*, *S. iactans*, *S. longipalis*, and *S. daghestanica*); Groups 11 and 13 each comprised two species (*S. trilineata* + *S. mathesoni* and *S. mathesoni* + *S. modesta*, respectively); Group 16 included four species (*S. limitata* + *S. semistriata* + *S. venusta* + *S. nigrolineata*); and Group 17 comprised three species (*S. stagnalis* + *S. mayri* + *S. selecta*). These mixed-species MOTUs indicate unresolved species boundaries and the presence of closely related or potentially lineages within *Sigara*, which require further evaluation using an integrative taxonomic framework incorporating morphological, molecular, and ecological evidence.

### 3.4. Climate Ecological Differentiation Revealed by RDA and Random Forest

Through an integrated analysis using two complementary approaches-redundancy analysis (RDA) and random forest-this study further revealed the environmental drivers underlying the geographical distribution of the 29 candidate species (MOTUs) of the genus *Sigara*. The adjusted R^2^ of the RDA model was 55.90%, indicating that the 12 selected environmental variables jointly explained more than half of the variation in distribution. Among individual-factor effects, isothermality exhibited the highest explanatory contribution, followed by temperature- and precipitation-related variables of warm and wet quarters. By comparison, elevation, aridity-index and frost-related variables showed relatively low marginal explanatory values.

**Figure 5:**
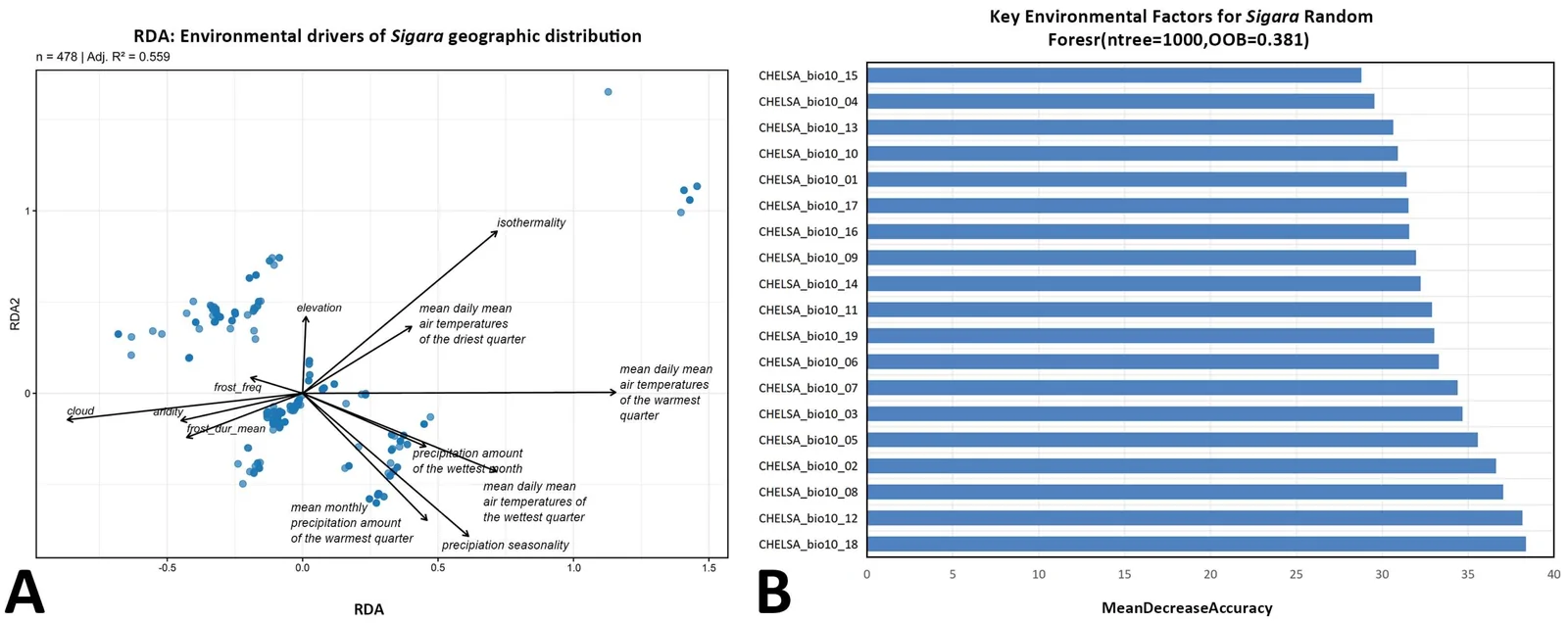
Ecological niche distribution patterns of *Sigara* species along environmental gradients. (**A**) PCA plot based on standardized environmental variables; (**B**) *Sigara* random forest importance assessment of environmental factors.

In the RDA biplot, the vectors of temperature and precipitation variables (isothermality, mean daily meanair temperatures, mean daily mean air temperatures of the driest quarter, mean daily mean air temperatures of the warmest quarter, mean monthly precipitation amount of the warmest quarter) are aggregated at an acute angle, indicating a temperature-precipitation synergistic gradient. Vectors representing frost frequency and mean frost duration pointed to the direction opposite to the major temperature-precipitation vectors on the RDA biplot. Despite this clear ordination-space separation, these frost-associated variables returned low marginal explanatory values in the RDA model. The altitude vector is extremely short, and its weak influence is confirmed again (Figure 5A).

The random forest model used 29 MOTUs as response variables and 19 CHELSA bioclimatic variables as predictors (ntree = 1000) (Figure 5B). The final out-of-bag (OOB) error was 0.381. Nevertheless, approximately 38% of the samples were misclassified, a proportion that was highly consistent with the uninterpreted variation in the RDA model. The importance of the variables is sorted by Mean Decrease Accuracy, and the top five are mean monthly precipitation amount of the warmest quarter (CHELSA_bio10_18, importance 38.35), annual precipitation amount (CHELSA_bio10_12, 38.16), mean daily meanair temperatures (CHELSA_bio10_08, 37.03), mean diurnal air temperature range (CHELSA_bio10_02, 36.63) and mean daily maximum air temperature of the warmest month (CHELSA_bio10_05, 35.56). These variables all belong to the warmest quarter temperature series, echoing the high contributions of mean monthly precipitation amount of the warmest quarter and mean daily mean air temperatures of the warmest quarter in the RDA analysis. There are differences in the ranking of variables between the two methods: RDA ranks isothermality as the first, reflecting its sensitivity to the proportion of diurnal and annual temperature changes; random forest is more dependent on the absolute temperature value, reflecting its ability to capture nonlinear interactions. This difference is complementary, suggesting that the interannual stability of temperature (isothermality) together with the absolute temperature and precipitation of the warmest quarter jointly constitute the multidimensional climatic niche underlying species differentiation in the genus *Sigara*.

In summary, the seasonal characteristics of temperature and the synchronization of summer water and heat are the dominant ecological filtering mechanisms controlling the geographical distribution of *Sigara*, while cold stress, drought and altitude only play local or marginal limiting roles.

## 4. Discussion

### 4.1. Species Boundaries by ABGD and NJ Analysis

The geographical distribution of the dataset showed substantial sampling bias, as Western Europe, North America, and East Asia contributed over 70% of the records, while Southern Europe, Northern Europe, and Oceania were poorly represented, likely due to uneven sampling intensity and differences in regional *Sigara* diversity. This imbalance is not unique to the *Sigara* study, but reflects the geographical sampling bias that is prevalent in current DNA barcoding studies [49]. This bias is not an isolated case. Bergsten et al. (2012) demonstrated that as the sampling scale expands from local to continental, the proportion of monophyletic species decreases significantly, with intraspecific variation being positively correlated with geographical scale (R^2^ = 0.7), and the success rate of identification also declines substantially [49,50,51,52]. Therefore, in the present study, the densely sampled regions may have overestimated intraspecific diversity, while the poorly covered regions may have severely underestimated the true number of species, indicating that future sampling efforts should be strengthened in regions such as Southern Europe, Northern Europe, and Oceania.

The dataset comprised a total of 46 BINs, of which 31 were concordant BINs and 15 were singleton BINs, overall revealing a clear barcode partitioning pattern. With a relaxed prior (P_max_ = 0.05), ABGD identified 29 MOTUs, while the NJ tree delimited 31 MOTUs with strong bootstrap support (>80%). Both methods largely corresponded with morphologically identified species, supporting the utility of *COI* barcoding for *Sigara* identification. The higher resolution of the NJ approach may result from its greater sensitivity to shallow genetic divergence, whereas ABGD tends to cluster closely related lineages with low genetic distances (<5%) [44,53].

However, both the NJ tree and ABGD analyses revealed that multiple species shared the same MOTU, a phenomenon particularly prominent in Group 2, which contained as many as eight morphologically defined species (*S. distincta*, *S. fossarum*, *S. falleni*, *S. scotti*, *S. fallenoidea*, *S. iactans*, *S. longipalis*, and *S. daghestanica*), indicating a significant discordance between molecular and morphological delimitation within this genus. Notably, it is worth noting that all morphologically verified species of the 95 new sequences identified in this study formed a monophyletic group, further confirming the high reliability of the *COI* barcode for species identification in *Sigara* [54]. However, some groups, such as *S. lateralis*, *S. truncatipala* and *S. nigrolineata* were split into multiple MOTUs, which may reflect misidentification or substantial genetic differentiation among cryptic species or geographically distinct populations [55]. The species (*S. selecta*, *S. stagnalis* and *S. mayri*; *S. venusta*, *S. semistriata*, *S. limitata* and *S. nigrolineata*; *S. falleni, S. longipalis, S. fallenoidea, S. distincta* and *S. iactans*) showed inter-embedded clade structure. These phenomena may be caused by species differentiation [21,56]. A barcoding study of German aquatic heteroptera insects by Havemann et al. (2018) [17] also found that about 18% of species share BIN, and the genetic distance between intraspecific and interspecific overlaps. Although *COI*-based analyses may be affected by the amplification of nuclear mitochondrial DNA pseudogenes (NUMTs), which have occasionally been amplified by the Folmer primers LCO1490 and HCO2198 in aquatic Heteroptera (e.g., *Hesperocorixa distanti*) [57], sequences with internal stop codons or poor quality were excluded before analysis. Nevertheless, NUMT amplification cannot be completely ruled out based solely on COI data. Therefore, the molecular diversity and MOTU patterns identified here should be interpreted with this limitation in mind.

### 4.2. Analysis of Environmental Driving Factors for the Genus Sigara

The analysis of environmental driving factors provides a more in-depth ecological explanation. The corrected interpretation rate of the RDA model is 55.9%, and the overall discrimination accuracy of the random forest is about 62% (1-OOB). The two are highly consistent, indicating that the selected climate and terrain variables can explain more than half of the distribution variation. The remaining 44% of the variation may be due to biological factors (such as competition, predation, and host plants) that are not included in the model, local microhabitat characteristics (water pH, conductivity, and aquatic vegetation coverage) and spatial historical processes (diffusion limits, geological historical events). 

The importance of temperature-related variables identified in both models is consistent with previous studies on Corixidae distribution. Barahona et al. (2005) [58] demonstrated that temperature exerts a significant effect on egg development and nymphal growth in *S. selecta*, with a minimum temperature threshold of 10 °C and a diel amplitude of ≥10 °C required for reproduction and oviposition. The present study extends these findings by quantifying the relative contributions of specific temperature metrics-particularly isothermality and warmest quarter temperature series-at a broader spatial scale.

However, the discrepancy in variable importance ranking between RDA and random forest merits further consideration. RDA identified isothermality (the ratio of diurnal temperature difference to annual temperature difference) as the primary driving factor, whereas random forest highlighted the warmest quarter temperature series (including mean monthly precipitation and annual precipitation of the warmest quarter) as most influential. This methodological divergence likely reflects the distinct statistical philosophies of the two approaches: RDA captures linear constraints on community composition, while random forest is more sensitive to threshold effects and nonlinear interactions among predictors. The complementary nature of these results suggests that *Sigara* species differentiation is governed by both the proportional structure of thermal seasonality (isothermality) and the absolute thermal intensity during critical reproductive periods (warmest quarter conditions). This dual climatic control aligns with the physiological findings of Carbonell et al. (2017) [59], who reported that marginal populations of *S. selecta* exhibit higher phenotypic plasticity in metabolic and reproductive traits in response to temperature variation, thereby facilitating persistence across thermally heterogeneous environments.

In addition, the moderate contribution of mean daily meanair temperatures and cloud amount in the wettest quarter suggested that water stability and light conditions were also involved in regulating the distribution pattern. The contribution of frost frequency and drought index is low, indicating that cold and drought are mainly used as boundary limiting factors of geographical distribution rather than fine adjustment factors. Elevation exhibited little independent contribution, suggesting that its apparent influence on *Sigara* distribution was largely captured by temperature-related variables.

Notably, Group 13 (n = 98) and Group 2 (n = 90) exhibited high mean values across multiple environmental variables and had a wide distribution range, which may correspond to generalist species with large niche breadth. In contrast, the rare groups (e.g., Group 15, 20, 22) had missing or abnormal environmental data, suggesting that these sequences may be derived from specimens without valid geographic coordinates or contain sequencing errors, and thus require further verification. In addition, approximately 40% of the variation remained unexplained by both RDA and random forest models, which indicates that future studies should incorporate higher-resolution local microhabitat variables (water depth, sediment substrate, aquatic vegetation, water physicochemical properties) and biotic interaction factors (competition, predation, parasitism).

## 5. Conclusions

In this study, a DNA barcode reference library for the genus *Sigara* was constructed, comprising 501 *COI* sequences from 15 countries. DNA barcoding revealed substantial lineage diversity and a clear barcode gap in *Sigara*, indicating distinct genetic structuring and potential cryptic diversity, while mixed and split MOTUs highlighted complex species boundaries and the need for broader geographic sampling and integrative taxonomic evaluation. Climatic analysis indicated that temperature seasonality (isothermality and warmest quarter temperature) is the dominant driver of distribution, with the models explaining approximately 60% of the variation, and the remaining variation may be attributed to local microhabitat conditions and biotic interactions. Affected by sampling bias, future efforts should strengthen sampling in the Southern Hemisphere and undersampled regions, and incorporate nuclear gene markers to refine species delimitation, thereby providing a reliable foundation for taxonomy, monitoring, and biogeography.

## Data Availability

A list of all species, specimens, their individual images, georeferences, primers, sequences, and other relevant laboratory data of all *Sigara* specimens are available through the public dataset “Global COI barcodes of *Sigara* (DS-SIGARA)” in the Barcode of Life Data System (http://www.boldsystems.org).

## Supplementary Materials

The following supporting information can be downloaded at: https://www.mdpi.com/article/10.3390/insects17080876/s1, Table S1. The Checklist of *Sigara* in the world; Table S2. *Sigara* specimens list with MOTUs (ABGD-based).
